# Development of a simultaneous bioreactor system for characterization of gas production kinetics of methanogenic archaea at high pressure

**DOI:** 10.1002/elsc.201900035

**Published:** 2019-05-31

**Authors:** Patricia Anna Pappenreiter, Sara Zwirtmayr, Lisa‐Maria Mauerhofer, Simon Karl‐Maria Rasso Rittmann, Christian Paulik

**Affiliations:** ^1^ Institute for Chemical Technology of Organic Materials Johannes Kepler University Linz Linz Austria; ^2^ Archaea Physiology & Biotechnology Group Archaea Biology and Ecogenomics Division Department of Ecogenomics and Systems Biology Universität Wien Wien Austria

**Keywords:** closed batch, high pressure, methanogens, microbiological physiology, parallel bioreactor

## Abstract

Cultivation of methanogens under high pressure offers a great opportunity in biotechnological processes, one of which is the improvement of the gas‐liquid transfer of substrate gases into the medium broth. This article describes a newly developed simultaneous bioreactor system consisting of four identical cultivation vessels suitable for investigation of microbial activity at pressures up to 50 bar and temperatures up to 145°C. Initial pressure studies at 10 and 50 bar of the autotrophic and hydrogenotrophic methanogens *Methanothermobacter marburgensis*, *Methanobacterium palustre*, and *Methanobacterium thermaggregans* were performed to evaluate the reproducibility of the system as well as to test the productivity of these strains. The strains were compared with respect to gas conversion (%), methane evolution rate (MER) (mmol L^‐1 ^h^−1^), turnover rate (h^−1^), and maximum conversion rate (*k*_min_) (bar h^−1^). A pressure drop that can be explained by the reaction stoichiometry showed that all tested strains were active under pressurized conditions. Our study sheds light on the production kinetics of methanogenic strains under high‐pressure conditions. In addition, the simultaneous bioreactor system is a suitable first step screening system for analyzing the substrate uptake and/or production kinetics of gas conversion and/or gas production processes for barophilic or barotolerant microbes.

**Abbreviations**
IU: inoculation unit
*k*_min_: conversion rate (bar h^−1^)
*M. marburgensis*: *Methanothermobacter marburgensis*
*M. palustre*: *Methanobacterium palustre*
*M. thermaggregans*: *Methanobacterium thermaggregans*
MER: methane evolution rate (mmol L^−1^ h^−1^)
MM: *M. marburgensis* medium
MM10: *M. marburgensis* 10 bar experiment
MP10: *M. palustre* 10 bar experiment
SBRS: simultaneous bioreactor system
*X*: biomass concentration (g L^−1^)

## 1 INTRODUCTION

Recent developments in the field of renewable biofuel production have raised interest in finding new bioprocessing routes as an alternative to fossil fuels. Waste to value approaches, also partially represented nowadays by the “power to gas” concept, reveal promising applications in terms of carbon capture and utilization. One of the ideas behind this concept is the re‐evaluation of off‐gas streams that contain CO_2_ and H_2_, the latter being obtained from processes using excess renewable electricity, for conversion into methane (CH_4_) 1, 2, 3, 4. One example is the CO_2_‐based biological CH_4_ production process 5, 6, 7, 8. Here, CO_2_‐type hydrogenotrophic methanogens from the domain of archaea utilize H_2_ and CO_2_ as a carbon and energy source to autocatalytically produce CH_4_, water (H_2_O), and biomass *X* (g L^−1^) according to Equation (1). In this case, the methanogenic archaea act as a biocatalyst. This process is dependent on both the CO_2_ (CUR) and the H_2_ (HUR) uptake rate. The CH_4_ production is described by the CH_4_ evolution rate (MER) and parallels the water production, which is calculated via the H_2_O evolution rate (WER). Formation of biomass during this process is dependent of the volumetric biomass production rate (*r*_(X)_) 8.

(1) $$\begin{array}{ccc} & & \mathrm{CUR}\cdot {\mathrm{CO}}_{2}+\mathrm{HUR}\cdot H_{2}\to \mathrm{MER}\cdot {\mathrm{CH}}_{4}+r_{\left(X\right)}\cdot X\\ & & \;+\;\mathrm{WER}\cdot H_{2}O \end{array}$$

In most of the existing bioprocesses, the carbon and/or energy source is contained in a liquid feed and therefore the organisms are liquid‐limited during growth, such as during fermentative ethanol production. In the case of CO_2_‐type hydrogenotrophic methanogens, gaseous substrates are converted into gaseous products. In these bioprocesses, kinetic limitations are dependent on the mass transfer of gaseous substrate(s) into the liquid phase where substrates become bioavailable 9.

Several studies have investigated the gas‐liquid mass transfer by changing variables such as sparger systems, gas flow rates, stirrer geometry, and stirrer speed 10, 11. However, conventional methods that suggest operating processes at increased stirring rates to improve gas transfer often show some limitations due to the sensitivity of the microorganism to shear stress 12.

Since the solubility of gases in aqueous fluids also correlates with the prevailing partial pressure in the gas phase, operating at elevated pressures to further increase the concentrations of H_2_ and CO_2_ in the cultivation medium has been discussed 13, 14, 15. Applying pressure instead of increasing the stirring rate was shown to reduce shear stress, and positively affects the gas‐liquid transfer in the process 12. The gas transfer rate in a bioreactor is dependent on two main variables: the volumetric mass transfer coefficient (k_L_a), and the gradient between the equilibrium and actual concentration of the gas in the liquid phase (C^*^_G_ ‐ C_G_). While k_L_a is primarily dependent on the agitation speed and gassing rate, the concentration gradient is influenced by the partial pressure of a gas in the gas phase. The partial pressure of a gas can be varied either by changing the concentration of the reactant gas or by changing the operation pressure in the reactor. Utilizing these modifications to increase the partial pressure will result in positively influencing the concentration gradient 16.

A large number of methanogens were originally isolated from deep‐sea regions where high hydrostatic pressure (pressure increases proportionally to depth and weight of a liquid) predominates (e.g. deep‐sea hydrothermal vents). Many microbes isolated from such environments display a positive barophilic and/or barotolerant response when cultured under pressure by increasing their specific growth rate 17, 18. Therefore, further investigations on CO_2_‐type hydrogenotrophic methanogens, cultivated under high pressure, either hydrostatic or hyperbaric (pressure obtained by gas pressure; p > p_atm_), could have a major impact on the development of new bioprocesses considering the pressure tolerance of these microbes 9, 19.

**PRACTICAL APPLICATION**

Here, a newly developed simultaneous bioreactor system (SBRS), which is suitable for the cultivation of microorganisms at elevated pressure <50 bar, is described. International microbial strain collections currently house a variety of hydrogenotrophic and autotrophic or carboxydotrophic methanogens that have not yet been tested for key industrial variables and growth productivity. As the interest in biomethane production processes at high pressure increased over the years, this system is highly valuable as a screening station for investigating the productivity of high pressure‐grown methanogenic archaea. The results of our study have implications for the utilization of methanogens in future high pressure processes, as the SBRS is a suitable system for analyzing the substrate uptake and/or production kinetics of gas conversion and/or gas production processes.

To develop new bioprocesses, preparatory characterization steps (i.e. investigations into required substrates, optimal growth temperature, pH) for a certain biocatalyst are necessary for subsequently optimizing a process in terms of high product yield or biomass formation. The characterization of the biocatalyst by means of screening, optimization, and modification of process parameters is usually performed in microtiter plates, serum bottles, Schott bottles or Erlenmeyer flasks 20, 21, 22. Serum bottles or Erlenmeyer flasks are vitreous and therefore only withstand slight overpressures (serum bottles *p*_max_ < 2.0 bar; Schott bottles *p*_max_ < 1.0 bar). Consequently, they are not suitable for the characterization of barotolerant and barophilic microorganisms. Pressure resistant stainless steel bioreactors are commercially available for high overpressure ranges (e.g. Büchi *p*_max_ < 350 bar; Sartorius, Thermo Fisher Scientific). Scaled down bioreactors at a milliliter scale reduce labor intensity, the amount of media, costs, and require less space than customary systems. Several different parallel miniature bioreactor systems have already been developed that enable high throughput screening by increasing the completion rate of necessary experiments 23, 24, 25, 26. The parallel bioreactor system DASGIP^®^ (Eppendorf AG, Germany) is applicable for batch, fed‐batch, and continuously operated experiments at atmospheric pressure. Parallel miniature bioreactors suitable for experiments at hydrostatic pressures have already been described in the literature 14, 27. However, to the authors' knowledge, high throughput screening systems, which are suitable for microbial gas conversion experiments at hyperbaric pressures, have not yet been published.

This work focuses on the development of a SBRS, which was developed to examine CO_2_‐type hydrogenotrophic methanogens at elevated pressure levels of up to 50 bar. As proof of principle, various mesophilic and thermophilic CO_2_‐type hydrogenotrophic methanogens were cultivated in the SBRS and used to evaluate the reproducibility of the system.

## 2 MATERIALS AND METHODS

### 2.1 Reactor concept

**Figure 1:**
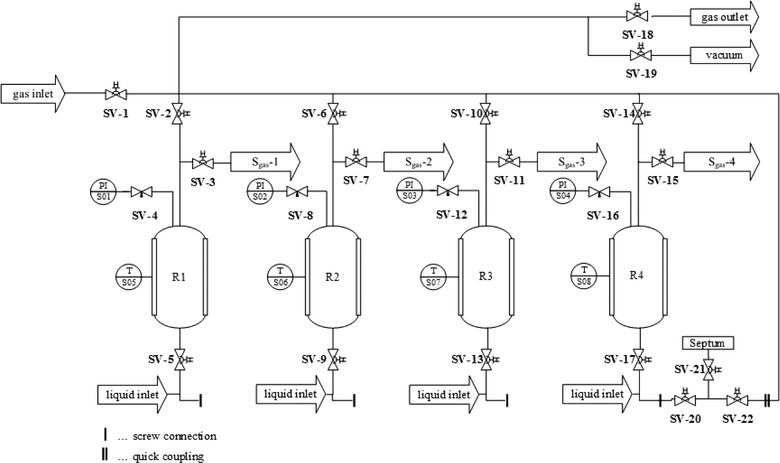
Piping and instrumentation diagram (DIN EN ISO 10628) of the SBRS. Each vessel (R1‐R4) is equipped with instrumentations such as pressure sensors (S01‐S04), heating jackets (S05‐S08), and gas lines including valves

The developed SBRS consists of four structurally identical bioreactors (R1‐R4) and enables simultaneous high‐pressure cultivations in closed batch cultivation mode (Figure 1). Each reactor has a total volume of 160 mL (diameter *d *= 42 mm, height *h *= 120 mm, wall thickness *x *= 2 mm) and can be operated independently. Each vessel is equipped with an individual heating jacket (S05‐S08; Keller Ihne Tesch GmbH, Austria, *d *= 42 mm, *h *= 120 mm) as well as a digital pressure sensor (Parker, 0‐60 bar, ± 5 V). The pressure sensors (S01–S04) are mounted on the top of the SBRS‐vessels and enable an online monitoring of pressure changes during the experiments. In addition to these pressure sensors, an analogous manometer (WIKA, 0–60 bar) was also installed. The bioreactors, the surrounding tubes, and the gas lines are made of stainless steel (1.4031, 1.4404) to avoid corrosion due to extreme conditions (e.g. high salt concentrations and corrosive gasses).

Data are collected by a data acquisition unit (ICP DAS, USB‐2019) and are recorded via LabVIEW (National Instruments; US). To increase gas/liquid mass transfer and to ensure liquid homogeneity, the SBRS is mounted on a horizontal laboratory shaker (SM‐B1, Edmund Bühler GmbH, Germany). The valves SV‐3, SV‐7, SV‐11 and SV‐15 serve as sampling ports for gas samples, whereas the gas inlet SV‐1 and gas outlet SV‐18 are used for pressurizing and depressurizing the whole system. For liquid supply, as well as draining liquid samples, the valves SV‐5, SV‐9, SV‐13 and SV‐17 are used.

### 2.2 Strains

The SBRS was tested with three different CO_2_‐type hydrogenotrophic methanogenic strains: *Methanothermobacter marburgensis* DSM 2133 (optimal growth temperature 65°C) 28, *Methanobacterium thermaggregans* DSM 3266 (optimal growth temperature 65°C) 29, and *Methanobacterium palustre* DSM 3108 (optimal growth temperature 37°C) 30. The methanogenic archaea strains were obtained from the Deutsche Stammsammlung für Mikroorganismen und Zellkulturen GmbH, Braunschweig, Germany.

### 2.3 Experimental set‐up

First, each SBRS vessel was filled with 120 mL of freshly prepared *M. marburgensis* medium (MM; 31). By applying a vacuum (SV‐19), subsequently flushing the vessels with a H_2_:CO_2_ gas mixture (80 Vol.‐% H_2_ in CO_2_; Air Liquide GmbH, Schwechat, Austria) via SV‐1, and by repeating this process four times, an anaerobic environment was established. Once an anaerobic environment was established, the vessels were closed off and autoclaved (20 min, 121°C). Following autoclaving, the temperature of each vessel was set to the optimum for the strain being grown (based on Deutsche Stammsammlung für Mikroorganismen und Zellkulturen GmbH data). The SBRS vessels were adjusted to the appropriate incubation pressure (9 or 49 bar depending on the experiments). During the experiments, the whole system was shaken by the laboratory shaker (ca. 70 min^−1^, horizontally). Once the preparation of the SBRS was concluded, the inoculation unit (IU), equipped with three ball valves (SV‐20, SV‐21, SV‐22, Figure 1) and a septum, was sterilized using a Certoclav (20 min, 121°C). The IU can easily be removed from the SBRS as it is attached with a screw connection to the vessel side and connects to the gas lines via quick coupling connections. The IU was flushed with a H_2_:CO_2_ gas mixture (approx. to 0.5 bar) while it was connected to the respective liquid inlet valve (SV‐5, SV‐9, SV‐13 or SV‐17). Prior to inoculation, each strain was flushed with a H_2_:CO_2_ (4:1) gas mixture and acclimated to room temperature. The inoculum (2.8 v/v %, 3.5 mL), along with a Na_2_S∙9H_2_O solution (0.5 mol L^‐1^, 1 v/v %, 1.25 mL) was injected through the septum into the IU by using a syringe. After SV‐21 was closed and the pressure in the IU was set to the initial inoculation pressure (10 or 50 bar), SV‐20 and the appropriate liquid inlet valve (SV‐5, SV‐9, SV‐13 or SV‐17) were opened and the cell suspension was flushed into the SBRS vessels by gas pressure.

### 2.4 Analytical techniques and assays

For taking gas samples the SBRS was equipped with the valves SV‐3, SV‐7, SV‐11, and SV‐15, where a cannula was screwed to each vessel. Since gas samples were stored in pre‐vacuumed glass serum bottles (120 mL; Macherey‐Nagel GmbH & Co.KG, Germany) only a residual pressure of max. 2.5 bar was allowed in the reactors to avoid bursting of the serum bottles due to overpressure. Sampling (gas and liquid) was only performed at the end of an experiment at low pressure conditions (max. 2.5 bar) in the SBRS (R1–R4). Biomass samples were simply obtained by opening SV‐5, SV‐9, SV‐13 or SV‐17. These samples were then centrifuged (4000 min^−1^; Thermo Fisher Scientific, US) and residual biomass was determined via the dry weight method (drying overnight, 105°C; Heraeus, US). The CH_4_ off‐gas concentration [Vol.‐%] of the obtained gas samples was determined twice with a gas chromatograph (Trace GC Ultra 2000, Thermo Fisher Scientific Inc., US) equipped with a thermo conductivity detector. Chromatographic separation was performed on a Carbonex‐1000 packed column (10 m, 3/8“). Injection volume was 1 mL of gas sample. The following GC parameters were used: inlet heater 150°C, detector 200°C, oven initial temperature 35°C, hold for 5 min, raise temperature at a rate of 20°C min^−1^ to 225°C, 10 min at final temperature. Helium served as the carrier gas with a constant pressure of 2.35 bar and a split flow of 90/10.

### 2.5 Data processing

Several studies in the past have used different parameters to describe CO_2_‐based biological methane production processes and evaluate the catalytic efficiency of methanogenic strains. For closed batch cultivations, Taubner et al. 2018 described an alternative way to indirectly quantify CH_4_ productivity by the turnover rate [h^−1^] (Equation (2)). This parameter, while neglecting biomass formation, is described by the difference in pressure before and after incubation Δ*p*, the maximum theoretical pressure difference due to stoichiometry Δ*p*_max_, and the time period Δ*t* of incubation 32.

(2) $$\mathrm{turnover}\;\mathrm{rate}\;\left[h^{-1}\right]\;=\frac{\Delta p}{\Delta p_{max}\Delta t}\;$$

Following the stoichiometry of methanogenesis, MER [mmol h^‐1^ L^‐1^] was calculated according to Equation (3) 33.

(3) $$\mathrm{MER}\;\;\left[mmolh^{-1}L^{-1}\right]=\frac{\Delta n_{CH4}}{\Delta t\cdot V}$$

The MER is defined by the difference in the number of millimoles of CH_4_ (Δn CH 4) per Δ*t* [h] and per total medium volume *V* [L]. As pressure data was recorded by online pressure sensors, the MER were calculated in intervals of 5 min between the individual data points. These MER values were used to determine the MER_max_ and further the respective point of time where MER reached its maximum. MER_total_ corresponds to the methane evolution rate calculated over the total experimental time. The *k* [bar h^−1^] was determined by calculating the slope between the individual data points, where the maximum negative slope *k*_min_ of the pressure curves is found at the inflection point using Equation (4).

(4) $$k\;\left[bar\;h^{-1}\right]\;=\frac{\Delta p}{\Delta t}\;$$

## 3 RESULTS AND DISCUSSION

*Methanothermobacter marburgensis* (MM10), *Methanobacterium palustre* (MP10), and *Methanobacterium thermaggregans* (MTa10) were cultivated at 10 bar in a 4:1 H_2_:CO_2_ mixture in quadruplicates for testing the reproducibility of gas conversion and gas production in the SBRS. In addition, the viability and activity of *M. thermaggregans* was tested at 50 bar (MTa50). Based on the stoichiometry of methanogenesis performed by CO_2_‐type hydrogenotrophic methanogens (Equation (1)), a pressure decrease in closed batch cultivation indicates gas conversion and is therefore a parameter for the activity of a methanogenic strain.

**Figure 2:**
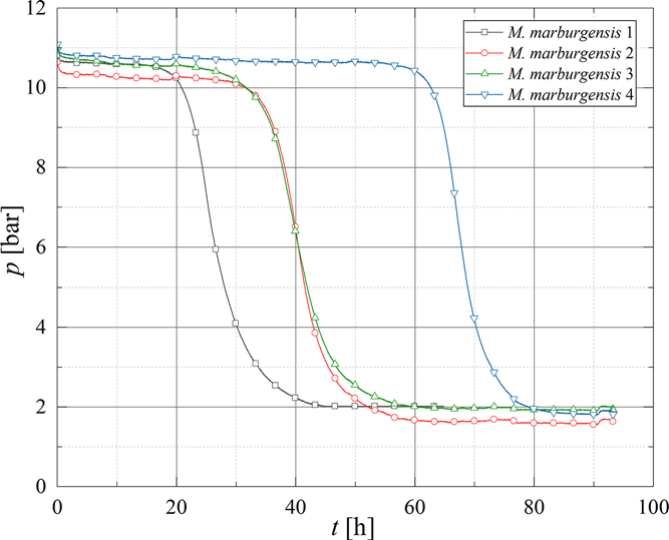
Pressure curves obtained during the gas conversion experiments with MM10 in the SBRS

**Figure 3:**
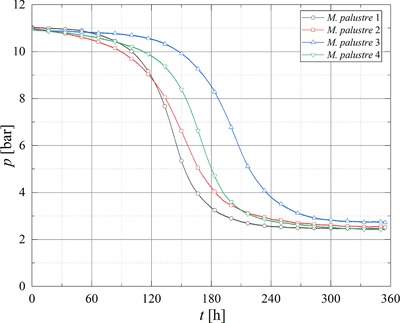
Pressure curves obtained during the gas conversion experiments with MP10

**Figure 4:**
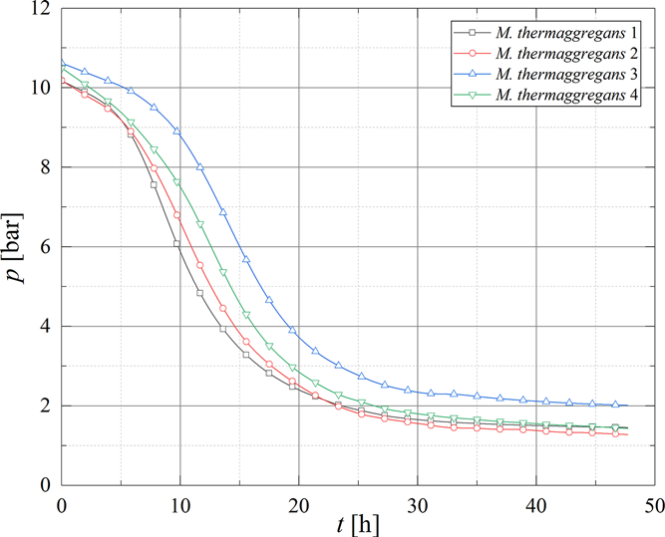
Pressure curves obtained during the gas conversion experiments with MTa10

A pressure drop was observed during cultivations of all strains, where the strains *M. thermaggregans* for both pressure levels and *M. marburgensis* showed conversions >94 %. However, for MP10 only a mean conversion of 87.87 (±1.32)% was observed (Table 1). The lag phases for *M. marburgensis* clearly varied (Figure 2), whereas *M. palustre* (Figure 3), and *M. thermaggregans* (Figure 4) showed reproducible gas conversion results when considering the length of the respective lag phases. The maximum conversion rate and the mean values for the CH_4_ off‐gas concentration, turnover rate, MER_total_, MER_max_, and the respective time at MER_max_ as well as *k*_min_ and the standard deviations were calculated from the data of the experiments performed in quadruplicates (Table 1). As the SBRS was equipped with online pressure sensors, the determination of MER and further the kinetics of the gas conversion could be performed more precisely. The amount of data points offered the opportunity to calculate MER every 5 min and further to determine MER_max_ in an experiment. In first step screenings of barophilic microorganisms, usually online sensors are not used and therefore such accurate studies of the gas conversion kinetics are not possible.

**Table 1:** Cultivation parameters and productivity (max. conversion rate, mean CH_4_ off‐gas concentration, turnover rate, MER_total_, MER_max_, *k_min_*) of tested methanogens

	*p*_inital_ bar	*p*_end_ bar	*T* °C	Conversion %	CH_4_% Vol.‐%	Turnover rate h^−1^	MER_total_ mmol h^−1^ L^−1^	MER_max_ mmol h^−1^ L^−1^	t_MER,max_ h	k_min_ bar h^−1^	t_kmin_ h
MM10	10.64 (±0.18)	1.84 (±0.16)	65	94.52 (±1.42)	99.04 (±0.56)	0.0150 (±0.0030)	0.459 (±0.125)	4.490 (±0.528)	42.40 (±15.49)	−0.931 (±0.120)	42.96 (±15.18)
MP10	10.96 (±0.06)	2.55 (±0.12)	37	87.87 (±1.32)	75.51 (±12.00)	0.0026 (±0.0002)	0.090 (±0.010)	1.004 (±0.102)	166.88 (±20.56)	−0.118 (±0.018)	166.28 (±23.36)
MTa10	10.37 (±0.20)	1.55 (±0.28)	65	97.02 (±2.69)	99.77 (±0.10)	0.0129 (±0.0004)	0.324 (±0.059)	2.807 (±0.193)	10.96 (±1.68)	−0.671 (±0.062)	11.45 (±2.03)
MTa50	48.95 (±0.86)	10.18 (±0.51)	65	96.36 (±0.81)	97.95 (±1.01)	0.0120 (±0.0014)	2.655 (±0.394)	11.927 (±2.103)	35.18 (±7.93)	−2.581 (±0.528)	35.63 (±8.28)

Data from dry weight determination is not shown because the amount of biomass gained from the liquid samples was insufficient to exactly determine the dry weight (>0.001 g) and outreached the measurable accuracy of the used analytical balance (VWR, LA214i).

**Figure 5:**
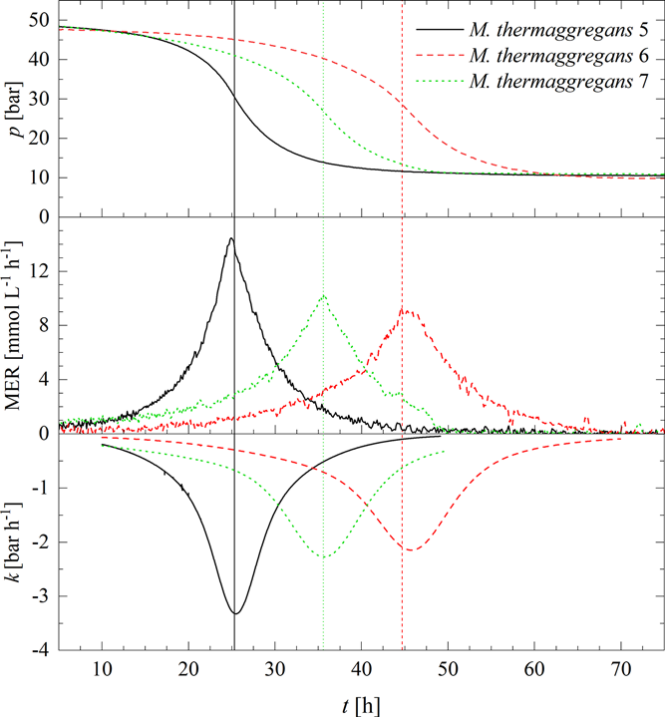
Pressure curves of MTa50 and the corresponding calculated MER and k values over time, where MER_max_ and k_min_ are determined by the peak maxima and peak minima respectively. Data is not shown for the replicate *M. thermaggregans* 8, because no pressure drop was detected)

In order to show that the maximum conversion rate during the gas conversion is reproducible, despite the differing lag phases, the maximum negative slope can be determined at the inflection point of the pressure curve. This maximum negative slope is indicated by *k*_min_ [bar h^‐1^]. Following the stoichiometry of autotrophic, hydrogenotrophic methanogenesis (Equation (1)), a pressure decrease will occur in a closed batch system. Additionally, with a steeper and decreasing slope, the *k* becomes more negative. Figure 5 illustrates the pressure curves for MTa50, the MER rate and slope *k* over time, where MER_max_ and *k*_min_ are indicated by the maxima and minima respectively. In the 10 bar experiments, MM10 shows the most negative *k*_min_ in contrast to MP10 and MTa10. Comparing the point of time, where MER_max_ and *k*_min_ is determined, *t*_MER,max_ and *t*_kmin_ show similar results, therefore both methods are suitable to calculate the maximum gas conversion activity of the strains in a closed batch system. Lemmer *et al*. 2017 investigated the methane production kinetics of methanogens cultivated on hydrolysate at high pressure, however a significant influence of the increase of the initial pressure on the increase of pressure was not observed 34. However, it is interesting to note that by comparing the results of MTa10 and MTa50 *k*_min_ becomes more negative. In this study, an unambiguous increase in MER_max_ as well as a decrease in *k*_min_ was observed, as MER_max_ increased from 2.807 (±0.193) to 11.927 (±2.103) mmol L^−1^ h^−1^ and *k*_min_ decreased from ‐0.671 (±0.062) to ‐2.581 (±0.528) bar h^−1^ when the incubation pressure was raised from 10 to 50 bar for *M. thermaggregans* (Table 1). It is therefore likely that these findings indicate a barophilic behavior of *M. thermaggregans* or an enhanced gas‐liquid transfer induced by the incubation pressure. Although, a correlation between an improved performance and an increased pressure was found for *M. thermaggregans*, this effect should not be assumed for other strains. Because barophilic behavior cannot be expected for other strains, further investigations are necessary. The turnover rate shown for MTa10 and MTa50 (Table 1) gives similar results. Nevertheless, the MER_total_ of MTa50 is 8x higher than the MER_total_ for MTa10 that can be explained by the higher available gas volume or pressure used in the MTa50 experiment (Table 1). In the 10 bar experiments, MP10 is the least efficient strain when turnover rate, conversion rate, MER_total_ and MER_max_ results are compared to the other tested thermophilic strains. *M. palustre* has an optimum growth temperature at 37°C and belongs to the group of mesophilic methanogens. According to the Arrhenius equation, higher temperatures generally accelerate chemical and enzymatic reactions and, therefore, specific growth rates (μ) and production rates. However, Sonnleitner and Fiechter 1983 disproved this dependency of μ within their studies on mesophiles and thermophiles. They found that the reliance between temperature and μ cannot be generalized for the production rates of mesophilic methanogens and *Bacilli*, as it might be assumed by the Arrhenius equation 35. Therefore, the results obtained and the behavior of *M. palustre* under overpressure should not be assumed for other mesophilic methanogens.

The simple structure of the SBRS creates clear advantages in the operation, and subsequently in the design, of high pressure experiments. The system is easily expandable by connecting additional independent reactors as only the gas supply and outlet lines are shared. Hence, the number of simultaneously performed experiments, and therefore output, can be increased. Since the SBRS was designed to characterize the barophilic behavior of methanogens, it is limited to closed batch cultivations and the monitoring of pressure changes at this stage of development. In general, the SBRS is not limited to cultivations of methanogens. It may also be suitable for studies involving other anaerobic organisms or processes, or even aerobic 14 microbes under high pressure that either use gases as an energy source or produce gaseous products.

## 4 CONCLUDING REMARKS

According to the results of this study, the newly developed SBRS is a suitable system for the rapid screening of barophilic or barotolerant CO_2_‐type hydrogenotrophic methanogens at hyperbaric pressures of 10 and 50 bar. As the system is suitable for a wide temperature range (25–145°C), screening of thermophilic and mesophilic strains is possible. Reproducibility was proven by cultivating three different strains at two different pressure levels, during which early information about the barophilic character and therefore the activity of the strains was obtained. The methanogenic archaea, *M. marburgensis, M. palustre*, and *M. thermaggregans* were successfully cultivated at 10 bar. *M. thermaggregans* was also successfully cultivated at 50 bar. CH_4_ production was achieved within all performed experiments. *M. thermaggregans* showed an increased conversion rate, turnover rate, MER_total_, and MER_max_ at 50 bar compared to 10 bar. *M. palustre* showed the lowest turnover rate, conversion rate, MER_total_, and MER_max_ in contrast to all other strains. It is concluded that the SBRS is a suitable system for analyzing the substrate uptake and/or production kinetics of gas conversion and/or gas production processes. The SBRS should serve as a simple set‐up to test barophilic or barotolerant strains and is not limited to experiments with anaerobic microorganisms.

## CONFLICT OF INTEREST

The authors have declared no conflict of interest.
